# Increases in Kratom-Related Reports to Poison Centers — National Poison Data System, United States, 2015–2025

**DOI:** 10.15585/mmwr.mm7511a1

**Published:** 2026-03-26

**Authors:** Eleanor Blair Towers, Ynhi T. Thomas, Christopher P. Holstege, Rita Farah

**Affiliations:** ^1^Department of Emergency Medicine, Division of Medical Toxicology, University of Virginia, Charlottesville, Virginia; ^2^Medical Scientist Training Program, University of Virginia, Charlottesville, Virginia; ^3^Henry J.N. Taub Department of Emergency Medicine, Baylor College of Medicine, Houston, Texas; ^4^Center for Innovations in Quality, Effectiveness, and Safety, Michael E. DeBakey Department of Veterans Affairs Medical Center, Houston, Texas.

**Summary**

**What is already known about this topic?** Kratom (*Mitragyna speciosa*), a plant used for its psychoactive properties, is widely available in the United States. The recent shift from traditional leaf preparations to high-potency alkaloid extracts has raised safety concerns.

**What is added by this report?**

Analysis of 2015–2025 National Poison Data System data found an increase of approximately 1,200% in kratom-related exposure reports (from 258 to 3,434), including a marked surge in 2025. Multiple-substance exposure reports, often involving addictive substances and antidepressants, were linked to the most severe clinical outcomes.

**What are the implications for public health practice?**

Kratom use remains a public health concern. Ongoing surveillance could help to identify high-risk patterns of use and guide public health education and clinical care, particularly for multisubstance use.

## Abstract

Kratom, the leaves of a tropical evergreen tree (*Mitragyna speciosa*), is traditionally consumed in Southeast Asia for pain relief, mood enhancement, and to relieve symptoms of opioid withdrawal. Kratom contains psychoactive compounds that interact with opioid receptors and is widely available in various forms in the United States. Its evolution from natural leaf to high-potency alkaloid products has raised concerns about toxicity. Data on kratom-related use that resulted in a report to the National Poison Data System (NPDS) (i.e., kratom exposure report) during 2015–2025 were analyzed to assess trends by exposure report type, demographic characteristics of persons exposed, and outcomes. During the past 11 years, poison centers received a total of 14,449 kratom exposure reports; the record high 3,434 reports in 2025 represent an increase of approximately 1,200% compared with the 258 reports in 2015. Most reports involved males and young adults aged 20–39 years, but reports among adults aged 40–59 years increased most sharply, with rates nearly overlapping with those among young adults by 2025. Although single-substance exposure reports accounted for most reports (62%), multiple-substance reports occurred at higher rates (range = 467–5,442 per 1 million multiple-substance drug exposure reports versus 388–4,045 per 1 million single-substance drug exposure reports), were associated with more hospitalizations (44%–56% versus 24%–29% annually) and serious (life threatening, pronounced, prolonged, or systemic) outcomes (57%–66% versus 41%–49% annually), and accounted for the vast majority of kratom-associated deaths during the study period (184 of 233; 79%). NPDS data indicate that kratom-related reports to poison centers are increasing and expanding among demographic groups, underscoring the value of ongoing surveillance to identify high-risk patterns of kratom use and guide strategies to reduce risks from multiple-substance exposure reports.

## Introduction

Kratom (*Mitragyna speciosa*), a Southeast Asian botanical, was historically consumed as crushed or brewed leaves for pain relief, mood enhancement, and to relieve opioid withdrawal symptoms (*1*,*2*). In the United States, kratom has shifted from these traditional preparations to a rapidly expanding commercial market of powders, tablets, gummies, and concentrated energy shots (*3*). This shift includes availability of high-potency products enriched with isolated kratom alkaloids, particularly 7-hydroxymitragynine, a *μ*-opioid receptor agonist that is marketed as kratom but is distinct from traditional kratom leaf preparations (*4*), prompting the Food and Drug Administration (FDA) to call for regulatory action focused on these products. Characterizing national patterns of kratom-related exposure reports can identify patterns of kratom exposures and determine which demographic groups are at highest risk for adverse effects. National Poison Data System (NPDS) data were analyzed to examine trends in kratom-related exposure reports, stratified by exposure report type (single- and multiple-substance exposure reports), demographic characteristics (i.e., age and sex), and medical outcomes.

## Methods

### Data Source and Study Period

Kratom exposure report data were extracted from NPDS, the data repository for 53 U.S. poison centers, for January 1, 2015–December 31, 2025. Each poison center submits, in near real-time, deidentified case data to NPDS after providing necessary poison exposure management and information services. The NPDS coding manual defines an exposure report as actual or suspected contact with a substance that prompted a consultation with a poison center, regardless of toxicity or clinical manifestations.

### Study Design and Case Identification

To align with the age groups used in the National Survey on Drug Use and Health, the primary U.S. substance use surveillance system, investigators queried NPDS for kratom exposure reports among persons aged ≥12 years. Both single-substance exposure reports (kratom as the only reported substance) and multiple-substance exposure reports (kratom and other substances reported for the same exposure) were included. No cases were excluded. Data on patient demographic characteristics (age and sex), exposure (substances and reported reason for use), level of care received, and medical outcome were included.

### Data Analysis

Data were analyzed by age group (12–19, 20–39, 40–59, and ≥60 years). Serious outcomes included exposures that resulted in death, major effects, and moderate effects.* Kratom-associated hospitalization was defined as an exposure that resulted in hospital admissions to critical care, noncritical care, or psychiatric units. For multiple-substance exposure reports, other substances were described by therapeutic class.^†^ Total kratom exposure report rates were calculated per 1 million drug exposure reports, single-substance kratom exposure report rates were calculated per 1 million single-substance drug exposure reports, and multiple-substance kratom exposure report rates were calculated per 1 million multiple-substance drug exposure reports. Analyses were stratified by exposure report type, sex, and age. Year-to-year changes in exposure report rates were examined using an exact two-sample Poisson rate-ratio test. The Benjamini–Hochberg procedure was used to adjust for multiple comparisons. The study was conducted using deidentified and publicly available data. The University of Virginia Institutional Review Board determined the research on deidentified and publicly available data did not require human subjects review.

## Results

### Exposure Report Trends

**FIGURE 1:**
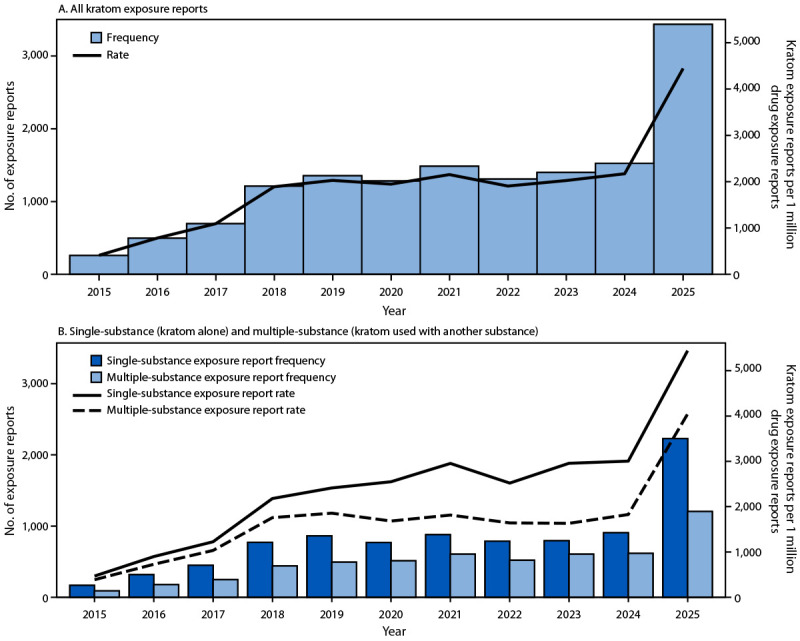
Frequency and rate* of kratom-related exposure reports to poison centers among persons aged ≥12 years, overall (A)^†^ and by single- or multiple-substance exposure report (B)^§^ — National Poison Data System, United States, 2015–2025 * Single-substance and multiple-substance kratom exposure reports per 1 million reported single-drug and multiple-drug exposure reports, respectively. ^†^ Year-to-year rates were significantly different during 2015–2018, 2020–2022, and 2024–2025. ^§^ Year-to-year rates were significantly different for single-substance exposure reports (kratom as the only substance reported) during 2015–2018 and 2023–2025 and multiple-substance exposures (kratom and another substance reported for the same exposure) during 2015–2018, 2020–2023, and 2024–2025.

During 2015–2025, U.S. poison centers documented 14,449 kratom exposures; the 3,434 exposures reported in 2025 represent an increase of approximately 1,200% compared with the 258 reported in 2015 (Figure 1). Exposure report rates increased in parallel with the number of exposure reports, from 412 to 4,445 per 1 million drug exposure reports, with a steady increase through 2019, a plateau during 2020–2024, and a marked surge in 2025 that exceeded all previous years (2,171 per 1 million drug exposure reports in 2024 compared with 4,445 per 1 million drug exposure reports in 2025) (Supplementary Table 1). During the 11-year study period, multiple-substance exposure reports (5,513), accounted for 38% of all kratom-associated exposure reports. Annual rates of multiple-substance exposure reports consistently exceeded rates of single-substance exposure reports (by 18%–81%); the 2025 rate of 5,442 per 1 million multiple-substance drug exposure reports was approximately 10 times higher than that in 2015 (467). The single-substance exposure report rate in 2025 (4,045 per 1 million single-substance drug exposure reports) was approximately nine times higher than the rate in 2015 (388).

Analyses by quarterly intervals demonstrated patterns similar to those observed in annual data (Supplementary Figure). The most common substances involved in multiple-substance exposure reports across this study period were addictive substances (ethanol, 22%; opioids, 16%; benzodiazepines, 15%; cannabis and cannabinoids, 12%; and stimulants, 11%) and antidepressants (14%).

### Demographic Characteristics of Persons with Kratom Exposure Reports

**FIGURE 2:**
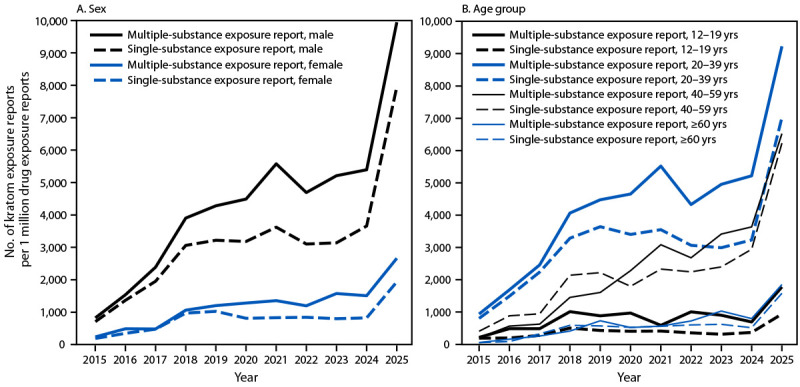
Rates* of kratom-related single- and multiple-substance exposure reports to poison centers among persons aged ≥12 years, by sex (A)^†,§^ and age group (B)^¶,^** — National Poison Data System, United States, 2015–2025 * Single-substance and multiple-substance kratom exposure reports per 1 million reported single-drug and multiple-drug exposure reports, respectively ^†^ Year-to-year rates were significantly different for single-substance exposure reports among males during 2015–2018, 2020–2022, 2023–2024, and 2024–2025 and among females during 2015–2018, 2019–2020, and 2024–2025. ^§^ Year-to-year rates were significantly different for multiple-substance exposure reports among males during 2015–2018, 2020–2022, and 2024–2025 and among females during 2015–2016, 2017–2018, 2022–2023, and 2024–2025. ^¶^ Year-to-year rates were significantly different for single-substance exposure reports among adolescents aged 12–19 years during 2017–2018 and 2024–2025; among adults aged 20–39 years during 2015–2018, 2021–2022, and 2024–2025; among adults aged 40–59 years during 2015–2016, 2017–2018, 2020–2021, and 2023–2025; and among adults aged ≥60 years during 2016–2018 and 2024–2025. ** Year-to-year rates were significantly different for multiple-substance exposure reports among adolescents aged 12–19 years during 2017–2018 and 2024–2025; among adults aged 20–39 years during 2015–2018, 2020–2022, and 2024–2025; among adults aged 40–59 years during 2015–2016, 2017–2018, 2019–2021, and 2024–2025; and among adults aged ≥60 years during 2024-2025.

During the study period, males accounted for the highest percentages of reported kratom-associated exposure reports (range = 65%–71% of single-substance and 67%–76% of multiple-substance exposure reports annually) and higher exposure report rates: 2025 rates of single-substance exposure among males (7,955 per million single-substance drug exposure reports) were 10 times higher than were those in 2015 (709), and multiple-substance rates in 2025 (9,945 per million multiple-substance drug exposure reports) were 11 times higher than were those in 2015 (825) (Figure 2). Among females, single-substance exposure reports rates in 2025 (1,937 per million single-substance drug exposure reports) were nine times higher than were rates in 2015 (190) and multiple-substance exposure report rates in 2025 (2,664 per million multiple-substance drug exposure reports) were 10 times higher than were those in 2015 (239).

### Level of Care and Medical Outcome

**FIGURE 3:**
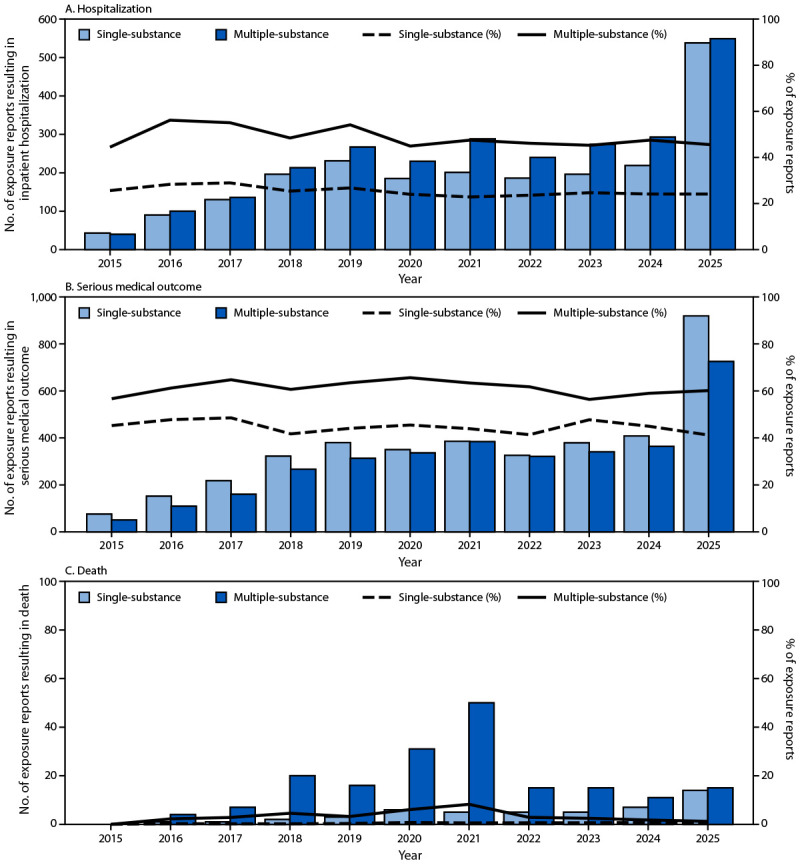
Frequency and percentage of kratom exposure reports resulting in critical care, noncritical care, or psychiatric hospitalization (A), serious medical outcomes* (B), and death (C) among persons aged ≥12 years reported to poison centers, by single- or multiple-substance exposure — National Poison Data System, United States, 2015–2025^†^ * Serious medical outcomes were defined as exposures resulting in death, major outcomes (those that are life-threatening or result in significant disability or disfigurement), or moderate outcomes (those that are pronounced, prolonged, or systemic, and usually require some form of treatment, but are not life-threatening), per National Poison Data System definitions. ^†^ Y-axis scales differ across panels.

Hospitalizations for single-substance kratom exposure reports increased 1,200%, from 43 in 2015 to 538 in 2025, and for multiple-substance exposure reports, increased 1,300%, from 40 to 549 (Figure 3). The annual percentage of hospitalizations was consistently higher among persons with multiple-substance exposure reports (44%–56%) than among those with single-substance exposure reports (24%–29%).

A similar pattern was observed for serious outcomes, which increased 1,100% for single-substance (from 76 in 2015 to 919 in 2025) and 1,300% for multiple-substance exposures reports (from 51 in 2015 to 725 in 2025), with consistently higher annual rates reported for multiple-substance exposure reports (57%–66%) than for single-substance exposure reports (41%–49%). Among 233 kratom-associated deaths reported during 2015–2025, which accounted for 3.2% of all 7,287 serious medical outcomes, 184 (79%) involved multiple substances. Opioids were reported in 62% of fatalities, followed by benzodiazepines (20%), stimulants (20%), and ethanol (19%) (Supplementary Table 2).

### Reason for Exposure Report

Intentional misuse was the most commonly reported reason for exposure report (56% of single-substance and 49% of multiple-substance exposure reports). Suspected suicide attempts were more frequent among persons with multiple-substance exposure reports (23%) than single-substance exposure reports (6%) (Supplementary Table 2).

## Discussion

During 2015–2025, kratom-related exposures reported to NPDS increased by approximately 1,200%, reaching record levels in 2025. Both single- and multiple-substance exposure reports increased during 2015–2019; thereafter, multiple-substance exposure reports continued to rise modestly through 2024, whereas single-substance exposure reports largely plateaued, with both increasing sharply in 2025. The large increase in 2025 coincides with the emergence of high-potency, semisynthetic formulations, including 7-hydroxymitragynine (*4*). National survey data among persons aged ≥12 years demonstrate that although annual kratom use prevalence was stable from 2019 to 2023, lifetime use increased from 4 million to 5 million persons, indicating that more persons are trying kratom (*5*). FDA import data further demonstrate record-high demand, as evidenced by 2025 FDA Import Alert 54–15, which addressed the high volume of kratom-containing products entering the United States (*6*). Consistent with these trends, this analysis found sharp increases in exposure reports among adults aged 40–59 years, with rates nearly matching those in persons aged 20–39 years. Together, these findings indicate that kratom use is increasing and expanding across demographic groups, underscoring a growing public health concern. In 2025, among all multiple-substance exposure reports, 60% resulted in serious medical outcomes and approximately one half required hospitalization. Enhanced surveillance and public health education could be beneficial given kratom’s widespread availability, lack of regulation, minimal medical oversight, and involvement in high-risk multisubstance exposures.

The findings in this report describe the impact of the rapidly evolving kratom market and highlight the important role poison centers can play as an early warning surveillance system to detect new trends and guide community partners, including clinicians, members of the public, and public health leadership. Severe outcomes were observed among persons who used kratom with other substances: approximately one half required inpatient hospitalization, and 79% of reported kratom-associated deaths involved multiple substances. Kratom use with alcohol, opioids, benzodiazepines, stimulants, and antidepressants might increase risk through additive pharmacodynamic effects on central nervous system pathways and through pharmacokinetic interactions that increase systemic exposure to substances used with kratom (*1*,*2*,*7*). This concern is heightened by the emergence of semisynthetic kratom products that have higher affinity for the opioid receptor, given that the majority of deaths involved kratom use with opioids (*4*). Psychiatric comorbidity might also compound harm. Previous studies indicate that approximately one third of kratom users met criteria for another substance use disorder (*8*), and approximately two thirds reported using kratom to manage depression or anxiety (*9*). Consistent with these findings, suicide attempts accounted for approximately one fourth of multiple-substance exposure reports, compared with only 6% of single-substance exposure reports, and antidepressants were involved in 14% of multiple-substance exposure reports, underscoring a connection between kratom use and mental health crises.

### Limitations

The findings in this report are subject to at least four limitations. First, NPDS relies on voluntary, self-reported data that might result in an underestimate of the number of milder events. Second, the poison center reports included reports from repeat callers, and certain substances or outcomes might have been misclassified despite standardized procedures. Third, the data do not include information about whether the kratom use involved traditional leaf products or semisynthetic or concentrated formulations, such as 7-hydroxymitragynine, thereby limiting conclusions about formulation-specific risks. Finally, multiple substances can be reported for each exposure report, and determining which substance was most related to clinical effects or medical outcome, including death, was not possible.

### Implications for Public Health Practice

Kratom-related adverse effects are increasing in number and complexity in the United States. Increasing use, the availability of high-potency kratom, and frequent multiple-substance exposure reports contribute to hospitalizations from physical as well as psychiatric causes. As FDA moves to regulate 7-hydroxymitragynine but not whole-leaf kratom products, surveillance should distinguish product types to assess risks (*10*). Building this evidence base is essential to promoting safe kratom use, identifying high-risk combinations of substances, and guiding public health action to prevent future health effects in this rapidly evolving drug landscape.

## Footnotes

* The medical outcome is the final determination made by the specialist in poison information, based upon all the information available at the conclusion of the case. NPDS defines major effects as life threatening or resulting in significant disability or disfigurement (e.g., status epilepticus). Moderate effects are pronounced, prolonged, or systemic and usually require some form of treatment, but are not life threatening (e.g., hypoglycemia with confusion). Minor effects are minimally bothersome and resolve rapidly (e.g., sinus tachycardia without hypotension). No effect is reserved for patients who develop no symptoms as a result of the exposure.

† Therapeutic classes included antidepressants (selective serotonin reuptake inhibitors, serotonin-norepinephrine reuptake inhibitors, tricyclic antidepressants, monoamine oxidase inhibitors, and other or unspecified antidepressants), stimulants (amphetamines and related compounds, cocaine, methamphetamine, methylphenidate, synthetic cathinones, synthetic phenethylamines, and other or unknown stimulants), cannabis and cannabinoid products (plant material, concentrates, edible products, cannabidiol, synthetic cannabinoids, and minor cannabinoids), and opioids (prescription opioids, heroin, buprenorphine, fentanyl, tramadol, and opioid combination products).
